# Potential of molecular based diagnostics and its impact on allergen immunotherapy

**DOI:** 10.1186/s40733-016-0024-8

**Published:** 2016-06-02

**Authors:** Giovanni Melioli, Eleonora Savi, Maria Angiola Crivellaro, Giovanni Passalacqua

**Affiliations:** 1 Allergy and Respiratory Diseases, Department of Internal Medicine, IRCCS San Martino-IST-University of Genoa, L. go R. Benzi 10, 16132 Genoa, Italy; 2 Allergology Unit, Ospedale A. Da Saliceto, Piacenza, Italy; 3 Allergology Service, Department of Medicine and Public Health, University of Padua, Padova, Italy; 4 Allergy and Respiratory Diseases, Department of Internal Medicine, IRCCS San Martino-IST-University of Genoa, Genova, Italy

**Keywords:** Molecular based diagnosis, Allergen Immunotherapy, Components, In vitro diagnosis

## Abstract

Molecular based in vitro technologies greatly changed the diagnostic approaches in allergy. At present, sensitization profiles can be dissected according to IgE subsets, which are specific for genuine or cross-reacting components and potentially dangerous or virtually harmless components. The identification of IgE in components with specific characteristics has a direct impact on the accuracy of the diagnosis (indeed, it is possible at present to not only identify the allergen derived from a given allergen source but also the family of molecules to which the patient is sensitized), on the prognosis of the patient’s allergy, and on the prevention activities to be implemented. More interestingly, during the last few years, and thanks to the tools of molecular diagnostics, the indications for Allergen Immunotherapy (AIT) have also be modified, and novel strategies for the selection of the allergens to be administered have been better defined. Indeed, protocols indicating how Molecular Based Diagnosis (MBD) can be used to identify the best AIT approach have been recently published. In this review, the rationale for the use of MBD tools is discussed, and the recent strategies for the choice of allergens to be used in AIT are reported.

## Background

Molecular based diagnosis (MBD) was introduced to “in vivo” and in “in vitro” allergy diagnostics 15 years ago [1], and its role is well defined at present [2, 3]. The use of single components derived from the complex mixture of proteins contained in the raw allergen extract allows us to obtain an accurate representation of the IgE sensitization profile. The IgE-mediated reactivity to specific components (i.e., proteins that are genuine sensitizers from a single allergen or allergen family) is suggestive of true sensitization. On the contrary, the presence of IgE directed to cross-reacting components (i.e., proteins able to bind IgE within a heterogeneous group of allergens) suggests sensitization to many different allergen sources, which at times is not strictly related in taxonomy [4]. All these aspects are currently part of the diagnostic approach [5].

MBD is particularly useful when Allergen Immunotherapy (AIT) [6] must be prescribed to patients with respiratory and hymenoptera allergies. An accurate MBD is substantially useful in patients with food allergies and, more importantly, in patients with pollen-food syndromes [7], especially when serum IgE directed to cross-reacting components are involved in the pathogenesis of the clinical disease [2]. With regards to food allergies, MBD is even more useful in the identification of food components that should be avoided in diet. In the light of these considerations, we will review herein the rationale for the use of MBD in prescribing AIT as well as when MBD can be profitably applied in clinical practice.

## The rationale for the use of MBD in the prescription of AIT

From a theoretical point of view, the more accurate the identification of the components evoking the IgE mediated response is, the most appropriate the prescription of the AIT will be. A paradigmatic example is represented by IgE sensitization to hymenoptera venoms [6]. In such a case, the allergens (as well as the relevant components) are well defined, the route of sensitization is unique, potentially cross-reacting components (such as carbohydrates CCD) are well known, and hymenoptera–allergic patients are generally mono-sensitized. Moreover, at risk occupations (e.g., beekeepers, gardeners) and the environment (rural areas, bee/wasp nests) strongly suggest or support the diagnosis. Additionally, highly specific reagents can be used for the diagnosis, and purified allergens can be used in therapy [8]. The situation may be different with regard to sensitization to common allergens such as grasses. Hay fever was originally described by Bostock in 1819, and the causal role of grass pollen was proven in 1873.

In grass sensitization, the period of pollination (May-July) usually correlates with symptoms, and symptoms only rarely occur outside this season.

The molecular analysis of grass pollen (in particular Phleum pratense derived proteins) showed that two major (Phl p 1 and Phl p 5), and some minor (Phl p 2, Phl p 4 etc.) components are detectable [9]. These items are “specific components” or genuine sensitizers. In other words, sensitization towards one or more of these components is associated with a true sensitization to Phleum pratense. Given that these proteins are related to the biological structure of grasses, many other plants can have similar profiles [10]. Thus, one could argue that AIT based on a single allergen that contains the most relevant component(s) should also protect from other allergens belonging to the same families [11, 12]. Different scenarios emerge when the IgE repertoire is represented by IgE against pan-allergens (e.g., Phl p 12 and Bet v 2, calcium binding proteins Phl p 7 and Bet v 4, and Pathogenesis Related proteins-10 Bet v 1, Cor a 1, Aln g 1). Immunological cross-reactivity is the result of the presence of widespread phylogenetically conserved proteins that show homologous epitopes [13] as well as the capacity of certain IgE to recognize, with different affinity, partially homologous proteins derived from different sources. It has been shown that cross-reacting components are not only less represented in AIT vaccines [14] but also that the IgE mediated immune-response to these components is weaker than that to major components [15–17]. Similar situations occur with mite and pet allergies.

Results of MBD were described in 2007 and offered an algorithm to identify which was the best combination of allergen(s) to be given [18]. More recently, it was shown that the best clinical results were obtained when AIT was given to patients sensitized to major components while unconvincing or controversial results were observed in patients sensitized to minor (or cross-reacting) components [19].

## The clinical experience in the use of MBD for the prescription of AIT

In recent years, many authors have noted that following an analysis of the IgE repertoire by MBD, the prescription of AIT could be modified. Sastre et al. [20] showed that in more than 50 % of patients, the allergen microarray results significantly changed the indication or the selection of allergens for immunotherapy. This finding is in agreement with the observations by Passalacqua et al. [15] AIT was firstly prescribed to 32 out of 318 poly-sensitized patients with respiratory symptoms. Following MBD, the prescription was changed in 3 of those patients by adding a new extract, and it was newly prescribed in 85 additional patients. In another study group, Moreno et al. [21] demonstrated that within a group of 1263 poly-sensitized patients 922 (73 %) would have been candidates for AIT to treat a mixture of grass and olive pollens based on available clinical data and skin prick test results. In 56.8 % of patients, there was non-coincidence in the composition of AIT that would be selected before and after investigators received the in vitro data.

In a large group of pediatric patients, Stringari [22] showed that after MBD, the SPT-based decision on AIT prescription or composition was changed in 277 (42 %) of 651 and 315 (48 %) of 651 children according to the European or American approach, respectively [23].

Some papers were then published suggesting strategies to define the composition of AIT starting from the MBD results (tailored AIT). One, in particular, is interesting because of the role of the glycidic chains of naturally derived highly purified components compared to standard recombinant *E. coli* derived components [24]. In this work, patients with IgE specific to recombinant genuine molecules, such as rPhl p 1, rPar j 2, and rOle e 1, are considered suitable for an AIT treatment because the reactivity to the component is specifically directed to the protein core of the allergen. On the contrary, when MBD results are obtained by the use of natural but highly purified glycoproteins, such as nCyn d 1, n Cup a 1 or nArt v 1, the presence of a cross-reactivity to CCD should be ruled out; it is only in this case that the suitability to AIT is accepted. In the same algorithm, a poor efficacy of AIT is expected in patients characterized by IgE that is directed to cross-reacting molecules such as profilins and polcalcins, while better results can be provided if these IgE specificities are absent.

Concerning the use of certain diagnostics tools to define AIT, the role of biomarkers is, at present, a new but strongly effective prospect in many other medical sciences, such as oncology or infectious diseases. For example, in a recent publication, FD Popescu revised potential biomarkers to be used in grass immunotherapy [25]. Many of these biomarkers can only be evaluated using research-based methodologies of study, but in the near future, it will be possible that a small but relevant panel of molecules will be available in clinical practice. From this point of view, AIT is the prototype of personalized medicine; as described in this review, many scientists are working to further personalize the treatment by the use of a more accurate analysis of the IgE repertoire of patients. If allergology is to follow the evolution of other medical sciences, biomarkers will be the next step to be considered and evaluated in controlled clinical trials before they are implemented in practice.

## Clinical practice

Could the large number of indications obtained by the above-mentioned works be translated into the allergists’ practice? At present, the answer is “yes” based on well-established data and “no” based on the uncertain aspects. Until now, no studies have been performed to the authors’ knowledge that have randomized patients’ AIT into two separate groups: one receiving an AIT prescription based on standard diagnosis and the other by adding MBD. In the second group in particular, rules to be used to define AIT should be similar those recently suggested [24]. In the absence of such a controlled study, the only evidence we have is the work of Schmid-Grendelmeier [19], who showed in an observational study that people with genuine sensitizations had better results than the group with IgE directed to cross-reacting components. One could argue that such a study has many intrinsic defects because it is observational and includes no defined control. Of course, an experimentally controlled and randomized study should be much more relevant to define the problem.

Despite these uncertain aspects, in the last 8 years, many authors have suggested indications regarding the strategy to be followed in prescribing AIT. Valenta et al. [18] indicated that MBD could support the optimal selection of pollinosis patients for AIT, at least in the Mediterranean area, and they also suggested what the central role of MBD is in monitoring the effects of AIT. The principle was simple and effective. MBD allows the identification of IgE directed to genuine components, such as Phl p 1 or Phl p 5, and to the cross-reacting components Phl p 7 and Phl p 12. The documentation of a genuine reactivity clearly indicates an AIT directed to those allergens. The documentation of an IgE reactivity against cross-reacting components not only explains the large number of allergens that resulted positive in SPT and IgE tests, but, in particular, it explains why AIT avoids allergens whose positivity was only due to a cross-reaction. These rules were further well defined by Moreno et al. [21]. With regards to the use of MBD in the follow-up, Sastre recently described that an adverse reaction can be associated with a certain “molecular” profile of patients treated with AIT [26].

Asero recently suggested to use Art v 1, Amb a 1, Par j 2, Bet v 1, Ole e 1, Pla l 1, Cup a 1, Phl p 1 and Phl p 5 as markers of primary sensitization to mugwort, ragweed, pellitory, birch, olive, plantain, cypress, and Phleum pratense pollens [13]. The presence of reactivity to these “genuine” components supports the indication to use AIT. This situation is easy to manage in the large number of cases in which one or a few allergens react with IgE. Nevertheless, in poly-sensitized patients, the situation could be much more complex [27]. To further indicate this complexity, the European and the American approaches should be carefully considered, as discussed recently [6]. In this context, the European school always suggests not to exceed the number of three different allergen sources for AIT in the same patient, while the American school treats patients with a mixture of all the positive allergens. Of course, advantages and disadvantages are apparent in these two approaches. In addition, it is evident that MBD was accepted enthusiastically and used by Europeans but was only barely recognized by Americans.

Special care should be paid in specific situations that are different from the previous examples. Even if the case is very rare, it is possible in a mite allergy that positive SPT and sIgE are associated with a pure sensitization to tropomyosins [28], a component family that shares allergens between mites, cockroaches, shrimps, snails, etc. AIT for mites does not contain Der p 10 (the mite tropomyosins) [29], and for this reason, its administration would be useless, at least if an immune reaction to tropomyosins is expected. Thus, the indication for the correct AIT is achieved by the collection of an accurate patient history and by the molecular assay with Der p 1, Der p 2, Der f 1, Der f 2 and Der p 10.

The introduction of the MBD is also changing the follow-up of treated patients. Indeed, it has been shown that severe reactions to olive AIT may occur in patients sensitized to Ole e 7 and Ole e 9 [30], and other similar reports are expected in the future.

Asero [13] and others [31] reported that AIT is equally effective in patients with single or multiple sensitizations if the administered allergen is carefully selected. Other authors have suggested simple and probably effective rules, although this observation has never been demonstrated with a properly designed clinical study. For example, Luengo stated that in the case of sensitization to the crude extract SPT and/or positive sIgE, AIT indication would be invisible if all components were negative because the extracts would be unlikely to contain the sensitizing molecule [32]. Finally, based on data from microarray allergen diagnostics (ISAC), Melioli et al identified 4 different clusters of patients with different reactivities [12]. The first was characterized only by a reactivity to genuine molecules, the second to genuine and few cross-reacting molecules, the third to both genuine and cross reacting molecules, and the fourth virtually only to cross-reacting reactive molecules. In an expert system to support the interpretation of ISAC results [33], these groups were implemented, and the potential capacity to be responsive to AIT is immediately calculated by the program. With regards to the use of allergen microarrays in clinics, too many concerns have slowed down their use in clinical practices, such as the high cost and the risk of unexpected results. In regards to the cost, a thorough investigation of the IgE profile using a single-plexed allergy diagnosis may be even more expensive in polysensitized patients. Unexpected results are mainly due to an unknown sensitization or to an incomplete collection of a patient’s history. However, many other relevant added values can be obtained from allergen microarrays. In particular, a clear picture of the sensitization profile is achieved, thus allowing the patient’s phenotyping; furthermore, the explanation of possible discrepancies between SPT or specific IgE results and ISAC are given. Then, multiplexed data allow the distinction between the sensitization to one or a few components of a cross-reacting allergen family and, in many cases, the identification of the first sensitizer is also suggested. Finally, an exhaustive IgE profile can be useful in pediatric or adolescent patients to follow-up the allergenic march at the molecular level [34].

An interesting and poorly explored context for molecular diagnosis is respiratory occupational allergy. Respiratory occupational allergy is a relevant problem, and it causes disabilities and socioeconomic consequences for both the patient and society. It is probably still underdiagnosed. A correct diagnosis is extremely important to reduce or limit the consequences of the disease. In any adult whose asthma or rhinitis begins or worsens while working, a diagnosis of respiratory occupational allergy should be considered, and a detailed occupational and medical history should be collected. All workers should be asked whether symptoms improve on days away from work or on holidays; positive answers should lead to further investigation. MBD helps to distinguish between sensitization to occupational exposures and cross-reactivities. Some important causes of occupational allergy are latex or wheat allergies. In a recent review, particular latex molecules [35] have been described to be major allergens in specific clinical phenotypes, such as in healthcare workers (Hev b 5 and Hev b 6), multi-operated patients (Hev b 11), subjects with spina bifida and patients with the so-called “latex –fruit syndrome” (Hev b 6). For example, several wheat proteins have been identified as causative allergens of occupational respiratory allergy [36] in bakery workers with wheat allergies (Tri a 14.). It is possible to test IgE reactivity in patients with different clinical profiles of wheat allergy (i.e., food allergy, wheat-dependent exercise-induced anaphylaxis, and baker’s asthma). In this regard, because immunotherapy could be attempted if allergen avoidance is not feasible, the aforementioned tool would allow a more precise diagnosis [37].

## Conclusions

In conclusion, even if we have no formal proofs, a considerable amount of significant evidence has been collected in recent years that shows that MBD has a direct effect on the strategy of choosing which allergens should be used in AIT-based clinical trials. At present, the application of this evidence is limited to the strategy of prescription based on European attitudes, but when regulatory authorities will ask for a more stringent documentation of the real IgE repertoire of the patients, MBD will find its ultimate role in the allergist armamentarium.

## Abbreviations

AIT: allergen immunotherapy
CCD: cross-reactive carbohydrate determinants
ISAC: immuno sorbent allergen chip
MBD: molecular based diagnosis
sIgE: specific IgE
SPT: skin prick test
